# Associations Between Interpersonal Problems, Negative Affect, and Symptoms of Night Eating Syndrome

**DOI:** 10.1002/eat.24463

**Published:** 2025-05-19

**Authors:** Charlotte P. H. Rottschäfer, Danielle Schewe, Martina de Zwaan, Bernhard Strauss, Elmar Brähler, Anja Hilbert

**Affiliations:** ^1^ Department of Psychosomatic Medicine and Psychotherapy, Behavioral Medicine Research Unit, Integrated Research and Treatment Center Adiposity Diseases Leipzig University Medical Center Leipzig Germany; ^2^ Department of Psychosomatic Medicine and Psychotherapy Hannover Medical School Hannover Germany; ^3^ Institute of Psychosocial Medicine, Psychotherapy and Psychooncology, University Hospital Jena, University of Jena Jena Germany; ^4^ Department of Psychosomatic Medicine and Psychotherapy University Medical Center of the Johannes Gutenberg‐University Mainz Germany; ^5^ Department of Psychiatry and Psychotherapy, Medical Faculty University of Leipzig Leipzig Germany

**Keywords:** insecure attachment, interpersonal problems, negative affect, night eating syndrome, social support

## Abstract

**Objective:**

Research on social support, attachment insecurity, and negative affect in night eating syndrome (NES) is sparse, although these factors have been proposed as key components of etiological models in other eating disorders. This study investigated whether individuals with night eating (NE) symptoms reported lower social support, greater attachment insecurity, and increased negative affect compared to those without, and examined if negative affect mediated the relationship between interpersonal problems (lack of social support, attachment insecurity) and NE symptoms.

**Method:**

A representative German population sample of 2423 participants (1297 women, 53.5%) aged between 18 and 92 years completed the Night Eating Questionnaire. Multivariate analysis of variance (MANOVA) analyzed differences between individuals with vs. without NE symptoms in social support, attachment insecurity, and negative affect. Mediation analyses examined cross‐sectionally negative affect as a mediator on the relationship between interpersonal problems (lacking social support and attachment insecurity) and NE symptoms.

**Results:**

Individuals with NE symptoms reported lower social support (less than small effect), more insecure attachment (small effect), and greater negative affect (small effect) than those without NE symptoms. Negative affect mediated the associations between social support or attachment insecurity and NE symptoms (small effect). All results remained significant after controlling for BMI.

**Discussion:**

Given elevated interpersonal problems and negative affect in individuals with vs. without NE symptoms, and the evidence of cross‐sectional applicability of the interpersonal model to NES, longitudinal research should examine this mediational effect and investigate interpersonal problems and negative affect as potential risk or maintaining factors of NES.

**Summary**

- Interpersonal problems and negative emotions are believed to contribute to eating disorders but are underexplored in night eating syndrome (NES).
- This study, using a representative German population sample, found that individuals with NE symptoms reported more interpersonal problems than those without, partly explained by increased negative emotions.
- As NES leads to lower quality of life, understanding its psychosocial factors is important to reduce stigmatization and enhance prevention and therapy approaches.

## 1 Introduction

Night eating syndrome (NES) is an eating disorder (ED) characterized by the core criteria of evening hyperphagia or nocturnal ingestions with an awareness of intake, leading to significant distress or impairment in functioning (Allison et al. 2010; American Psychiatric Association 2022). In the population, night eating (NE) symptoms occur with a prevalence between 0.5% and 1.5% (Muscatello et al. 2022) across sexes, age groups (de Zwaan et al. 2014), and ethnicities (Striegel‐Moore et al. 2006). NE symptoms are associated with a range of serious physical and mental health issues, including obesity (Lent et al. 2022; Tholin et al. 2009), increased depression and anxiety symptoms (Matsui et al. 2021), and impaired quality of life (Kim, Ju, et al. 2023). The onset of NES may be related to interpersonal problems (Cleator et al. 2014; Shillito et al. 2018) and negative affect (Cleator et al. 2014; Latzer et al. 2024), which are central components of widely used etiological models across other EDs (Rieger et al. 2010; Wilfley et al. 2000). However, only preliminary research has explored the role of interpersonal problems such as lacking social support (Shillito et al. 2018) and insecure attachment (Wilkinson et al. 2022), and negative affect (Cleator et al. 2014) in NES.

Social support, a general resistance resource facilitating stress management and emotional regulation (Antonovsky et al. 1997), was proposed as a coping mechanism in a qualitative study with 10 outpatients with NE symptoms (Shillito et al. 2018). In a second qualitative study with a clinical sample of 31 adults with NE symptoms, bullying and family conflict were identified as potential causes for the onset of NES (Cleator et al. 2014), which may indicate lacking social support. Apart from the sparse research on social support in NES, lower social support was cross‐sectionally associated with more binge eating, highly comorbid with NES (Lavery and Frum‐Vassallo 2022), in 2856 individuals from the population (Bentley et al. 2014). Moreover, lower social support was reported in adults with vs. without ED symptoms in a population sample of 36,309 adults (Kim, Smith, et al. 2023). Besides social support, secure attachment was proposed to enhance resilience and mental health (Bowlby 1988). Heightened levels of attachment anxiety were associated with NE symptoms in a clinical sample of 741 adults (Morse et al. 2006) and two population samples of 276 and 486 individuals (Wilkinson et al. 2022). In 370 adults with binge‐eating disorder (BED; American Psychiatric Association 2022), greater insecure attachment was found compared to a non‐clinical control group (Chyurlia et al. 2019). Attachment insecurity was also associated with ED symptoms in 921 non‐clinical adults (Roithmeier et al. 2024). These findings support the relevance of social support and attachment security in other EDs and the necessity for exploring their role in NES.

The interpersonal model of binge eating (Wilfley et al. 2000) proposes that negative affect mediates the relationship between interpersonal problems and binge eating, which was empirically supported in a cross‐sectional study of 255 women with BED (Ivanova et al. 2015). In NES, negative affect was proposed as a maintenance factor in qualitative studies of 31 adult outpatients (Cleator et al. 2014) and 18 women (Latzer et al. 2024) with NE symptoms. Furthermore, in two population‐based studies with 8348 (Matsui et al. 2021) and 34,359 adults (Kim et al. 2022), individuals with NE symptoms showed more symptoms of depression than participants without NE symptoms. Consistently, a network analysis of a community sample of 144 adults with NES identified depressed mood as a central feature of a NES psychopathology network (Beauchamp et al. 2021). Despite clinical differences between binge eating and NE symptoms, including a sense of loss of control over eating and usually greater body‐related concerns in individuals with binge eating than in those with NE symptoms (Kaur et al. 2022), a high comorbidity is described (Lavery and Frum‐Vassallo 2022). However, the proposed mediating effect of negative affect in BED (Ivanova et al. 2015; Wilfley et al. 2000) remains to be investigated in NES.

In this context, the present study aimed to investigate whether individuals with NE symptoms report lower social support, greater attachment insecurity, and more negative affect than individuals without NE symptoms in a large population‐based sample. Additionally, the mediating effect of negative affect on the relationships between social support and attachment insecurity with NE symptoms was investigated, expecting lower social support and greater attachment insecurity to be positively associated with higher negative affect, which in turn would be positively associated with NE symptoms.

## 2 Methods

### 2.1 Participants and Procedure

A cross‐sectional sample of the general German population, representative in terms of age, sex, and education, was selected for this study with the assistance of an independent institute for opinion and social research (USUMA, Berlin).

Initially, 258 nonoverlapping inhabited areas in Germany were randomly chosen according to the BIK Aschpurwis + Behrens GmbH classification system, which measures the degree of urbanization and geographical distribution. Utilizing the random‐route method, 4386 households were contacted, and target persons were selected with a Kish selection grid (Kish 1949). A total of 198 trained research assistants visited 4360 households, resulting in 2526 assessments after refusals and noncontacts. All assessed variables are displayed in Tables S1 and S2 in the Supporting Information. Participants were required to meet inclusion criteria (age ≥ 14 years, ability to understand written German language) and to provide oral informed consent. After excluding 18 nonvaluable assessments, a sample of 2508 individuals remained. In this study, participants were included if they were aged ≥ 18 years and had ≤ 25% missing data on the Night Eating Questionnaire (NEQ; Allison, Lundgren, et al. 2008), resulting in the final sample of 2423 participants (1297 women, 53.5%), aged between 18 and 92 years (*M* = 50.82, SD = 17.50), with a mean body mass index (BMI; kg/m^2^) of 25.32 kg/m^2^ (SD = 3.82; see Table 1 for sociodemographic and descriptive statistics). For sensitivity analysis, descriptive and correlative data of underage participants are reported in Tables S3 and S4 in the Supporting Information.

**TABLE 1:** Sociodemographic and descriptive statistics for the total sample and individuals with vs. without NE symptoms.

Sociodemographics	Missing values	Total sample, *N* = 2423		NE symptoms, *n* = 28		No NE symptoms, *n* = 2395				
	*n*	*n*	%	*n*	%	*n*	%	*U/ χ* ^*2*^	*Z/φ*	*p*
Sex, female	0	1297	53.5	20	71.4	1277	53.3	3.65	0.04	0.06
Nationality German Not German	0	2329 94	96.1 3.9	28 0	100 0	2301 94	96.1 3.9	1.14	−0.02	0.29
Years of school education ≥ 12 years	7	454	18.7	5	17.9	449	18.7	0.02	−0.00	0.89
Household income, €/month Low, < 900 Medium, 900–1999 High, ≥ 2000	71	182 1056 1114	7.5 43.6 46.0	9 8 10	32.1 28.6 35.7	173 1048 1104	7.2 43.8 46.1	24,084.50	−2.32	0.02
Family status Single Married/living together Married/not living together Divorced Widowed	0	622 1112 63 351 275	25.7 45.9 2.6 14.5 11.3	11 9 0 6 2	39.3 32.1 0 21.4 7.1	611 1103 63 345 273	25.5 46.1 2.6 14.4 11.4	5.33	0.05	0.26
Weight status, kg/m^2^ Underweight, < 18.5 Normal weight, 18.5–24.9 Overweight, 25–29.9 Obesity, ≥ 30	14	19 1162 994 234	0.8 48.0 41.0 9.7	0 5 15 8	0 17.9 53.6 28.6	19 1157 979 226	0.8 48.3 40.9 9.4	20,554.00	−3.87	< 0.001

		*M*	SD	*M*	SD	*M*	SD	*U*	*Z*	*p*
Age, years	0	50.82	17.50	44.54	16.39	50.89	17.50	26,455.00	−1.92	0.06
BMI, kg/m^2^	14	25.32	3.82	29.10	6.73	25.28	3.75	19,638.00	−3.74	< 0.001
F‐SozU K‐6, 1–30	12	23.98	4.60	21.41	4.00	24.01	4.58	20,668.00	−3.21	0.001
ECR‐RD12, 1–7	43	2.50	1.06	3.63	0.84	2.48	1.05	12,550.00	−5.18	< 0.001
PHQ‐4, 0–12	29	5.76	2.30	9.71	3.18	5.71	2.24	10,243.00	−6.59	< 0.001

*Note:* The NEQ cutoff categorized NE symptoms (≥ 25) and No NE symptoms (< 25). NEQ = Night Eating Questionnaire (0–52). Due to missing data, values may not sum up to 100%. Mann–Whitney *U* tests indicated differences between individuals with vs. without NE symptoms in ordinal and metric variables, whereas chi‐squared test investigated group differences in dichotomic and nominal variables. The significance level for these tests was *α* = 0.004 using Bonferroni correction.

Abbreviations: BMI = body mass index (kg/m^2^), ECR‐RD12 = Experiences in Close Relationships Questionnaire, F‐SozU K‐6 = Perceived Social Support Questionnaire, NES = night eating syndrome, PHQ‐4 = Patient Health Questionnaire‐4.

### 2.2 Measures

#### 2.2.1 NEQ

NE symptoms were assessed using the German version of the NEQ (Allison, Lundgren, et al. 2008; Meule et al. 2014), a 14‐item self‐report questionnaire with four subscales: evening hyperphagia (3 items), nocturnal ingestion (5 items), morning anorexia (2 items), and mood/insomnia (2/1 items). Item 13, addressing the awareness of intake, was not included in NEQ total score (Allison, Lundgren, et al. 2008). All items were rated on a 5‐point Likert scale, with most items scaled from 0 = “not at all”/“never” to 4 = “very much”/“completely”/“always.” Evening hyperphagia was defined by scoring > 1 in item 5, representing > 25% of daily intake after dinner, and nocturnal ingestion by scoring > 1 in item 12, indicating eating during at least half of the nocturnal awakenings (Allison, Engel, et al. 2008). The NEQ total score is a sum score, ranging between 0 and 52, with higher NEQ total scores indicating higher severity of NE symptoms. In this study, the widely used cutoff ≥ 25 defines the presence of NE symptoms, though the utilization of cutoff ≥ 30 is also discussed in NES research (Allison, Lundgren, et al. 2008). The total score demonstrated a reliability of McDonalds *ω* = 0.68 and Cronbach's *α* = 0.59 in this study's sample, consistent with prior psychometric research (Allison, Lundgren, et al. 2008; Meule et al. 2014).

#### 2.2.2 Perceived Social Support Questionnaire (F‐SozU K‐6)

To assess perceived social support, the six‐item version of the F‐SozU K‐6 (Kliem et al. 2015), adapted from the original 54‐item questionnaire (Sommer and Fydrich 1991), was used. Response options ranged from 1 = “does not apply” to 5 = “applies exactly.” Higher F‐SozU K‐6 sum scores indicate a higher level of perceived social support. The reliability of the F‐SozU K‐6 sum score was McDonalds *ω* = 0.89 and Cronbach's *α* = 0.89 in this study's sample.

#### 2.2.3 Experience in Close Relationships Revised Deutsch (ECR‐RD12)

The 12‐item ECR‐RD12 (Brenk‐Franz et al. 2018), derived from the ECR (Brennan et al. 1998), assessed adult attachment insecurity on a 7‐point Likert scale from 1 = “strongly disagree” to 7 = “strongly agree.” The self‐report questionnaire contains two subscales of attachment anxiety and avoidance with six items each. A higher ECR‐RD12 mean score indicates greater attachment insecurity. In this study's sample the ECR‐RD12 mean score showed an internal consistency of McDonalds *ω* = 0.80 and Cronbach's *α* = 0.85.

#### 2.2.4 Patient Health Questionnaire‐4 (PHQ‐4)

Negative affect was assessed with the PHQ‐4 (Löwe et al. 2010; Wicke et al. 2022), consisting of two depression items of the Patient Health Questionnaire‐2 (PHQ‐2; Kroenke et al. 2003) and two anxiety items of the Generalized Anxiety Disorder Scale (GAD‐2; Kroenke et al. 2007). Participants rated the frequency of depression and anxiety symptoms on a scale from 0 = “never” to 3 = “nearly every day.” Higher PHQ‐4 sum scores indicate more depression and anxiety symptoms. The reliability of the PHQ‐4 score was McDonalds *ω* = 0.88 and Cronbach's *α* = 0.88.

#### 2.2.5 Sociodemographics and Anthropometrics

Age, sex (male, female), nationality (German, not German), education (≥ 12 years, < 12 years of school education), and family status (single, married/living together, married/not living together, divorced, widowed) were assessed via self‐report. Self‐reported household net income was classified by the median income of the survey year into low (< 900€/month), medium (900–1999€/month), and high (≥ 2000€/month). BMI (kg/m^2^) was calculated from self‐reported weight and height.

### 2.3 Data Analytic Plan

Data were analyzed using IBM SPSS Statistics (Version 29). Missing values of the questionnaires were replaced with estimated individuals mean scores if ≥ 75% of items were completed. Internal consistency was assessed using the well‐established Cronbach's *α* (Cronbach 1951), assuming tau‐equivalence, and the potentially more accurate McDonald's *ω* (McDonald 1999), based on factor loadings.

To test assumptions of subsequent analyses and to evaluate potential covariates, associations between NE symptoms, social support, attachment insecurity, negative affect, and sociodemographic variables were examined using Spearman's correlation analyses. Spearman's rank correlation coefficient *ρ* was used for the interpretation of effect sizes as small (0.1 ≤ *ρ* < 0.3), medium (0.3 ≤ *ρ* < 0.5), and large (*ρ* ≥ 0.5; Cohen 1988) and a Bonferroni‐corrected significance level of *α* = 0.0014 was applied. Further, Mann–Whitney *U* tests were used to analyze differences between individuals with vs. without NE symptoms in ordinal and metric variables (household net income, weight status, age, BMI, and questionnaire mean scores), while chi‐squared tests examined group differences in dichotomic and nominal variables (sex, nationality, education, and family status), with a Bonferroni‐corrected significance level of *α* = 0.004. Covariates were selected if sociodemographic variables showed at least a medium size correlation with NE symptoms or if there were significant differences between individuals with vs. without NE symptoms.

MANOVA was employed to test for differences between individuals with and without NE symptoms in social support (F‐SozU K6), attachment insecurity (ECR‐RD12), and negative affect (PHQ‐4), with subsequent univariate analyses (ANOVAs) in case of a significant multivariate effect. Partial *η* ^2^ was reported as the effect size of ANOVAs, categorized as small (≥ 0.01 *η*_p_^2^ < 0.06), medium (≥ 0.06 < *η*_p_^2^ < 0.14), and large (*η*_p_^2^ ≥ 0.14; Cohen 1988). As NEQ cutoff ≥ 30 has also been discussed as an indication of NE symptoms (Allison, Lundgren, et al. 2008), a sensitivity analysis with NEQ cutoff ≥ 30 was calculated (see Table S5 and Supporting Information text).

**FIGURE 1:**
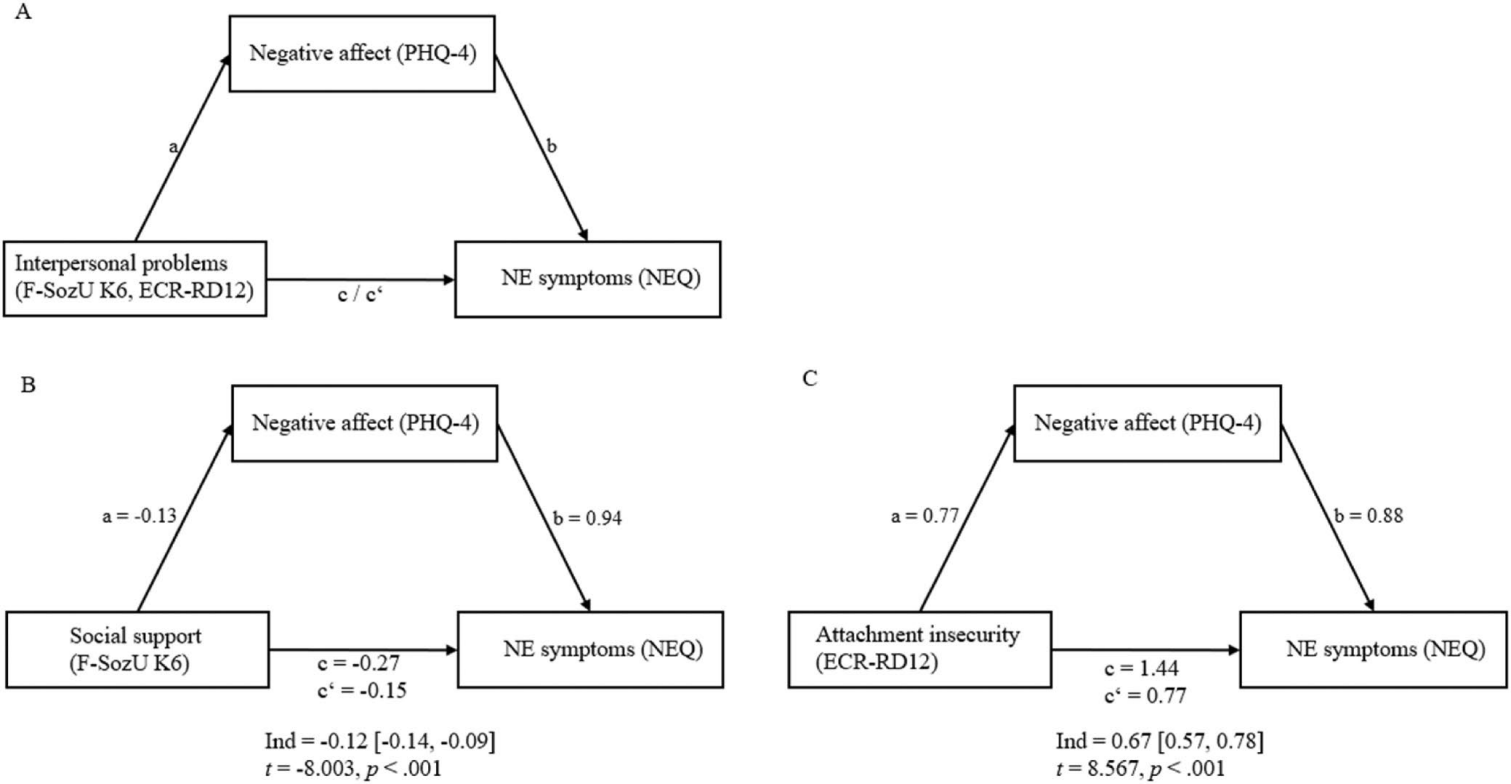
Model of negative affect as mediator between interpersonal problems and NE symptoms. All standardized coefficients are significant at two‐tailed *p* < 0.001, controlled for BMI. (A) Conceptional mediation model. (B) Standardized coefficients and indirect effect of the mediation of negative affect on social support and NE symptoms. (C) Standardized coefficients and indirect effect of the mediation of negative affect on attachment insecurity and NE symptoms. ECR‐RD12 = Experiences in Close Relationships Questionnaire, F‐SozU K‐6 = Perceived Social Support Questionnaire, NES = night eating syndrome, NEQ = Night Eating Questionnaire, PHQ‐4 = Patient Health Questionnaire.

Model 4 of the PROCESS macro by Hayes (2018) was used to examine the potential mediating effect of negative affect (PHQ‐4) on the relationship between interpersonal problems, operationalized as lacking social support (F‐SozU K6) or attachment insecurity (ECR‐RD12), and NE symptoms (NEQ). To estimate the indirect effects, bootstrapping with 5000 resamples was applied, with significance determined by 95% confidence intervals (CIs). The hypothesized model is shown in Figure 1. As effect size *R* ^2^ was used and interpreted as small (≤ 0.01 < *R* ^2^ < 0.13), medium (≤ 0.13 < *R* ^2^ < 0.26), and large (*R* ^2^ ≥ 0.26; Cohen 1988). For all analyses, a two‐tailed significance level of *α* < 0.05 was applied.

## 3 Results

### 3.1 Sample Characteristics and Correlations

As displayed in Table 1, 28 (1.03%) out of 2423 participants screened positive for NE symptoms. Among these, 12 (42.9%) individuals reported evening hyperphagia and 3 (10.7%) individuals nocturnal ingestion, of which one (3.6%) participant described both. Participants with NE symptoms were predominantly female, middle‐aged, overweight, and held German nationality. Further, individuals with NE symptoms had significantly lower social support, higher attachment insecurity and negative affect, and greater BMI than individuals without NE symptoms. NE symptoms correlated positively with attachment insecurity (medium effect), negative affect (medium effect), female sex, and BMI. A negative correlation was demonstrated between NE symptoms and social support (small effect), as well as school education (Table S6, Supporting Information materials). Since no sociodemographic variable showed a correlation with NE symptoms of at least medium effect size, but BMI differed significantly between individuals with vs. without NE symptoms, only BMI was included as a covariate in subsequent analyses.

### 3.2 Group Differences in Participants With and Without NE Symptoms

There was a significant multivariate effect of individuals with vs. without NE symptoms on social support, attachment insecurity, and negative affect, *F*(3, 234) = 27.373, *p* < 0.001. Post hoc univariate analyses revealed statistically significant differences between participants with and without NE symptoms in social support, *F*(1, 480) = 6.114, *p* = 0.01, *η*_p_^2^ = 0.003, less than small effect, attachment insecurity, *F*(1, 259) = 30.234, *p* < 0.001, *η*_p_^2^ = 0.013, small effect, and negative affect, *F*(1, 115) = 74.259, *p* < 0.001, *η*_p_^2^ = 0.031, small effect. Participants with NE symptoms reported lower social support, more insecure attachment, and greater negative affect compared to participants without NE symptoms. After adding BMI as a covariate, the multivariate analysis of covariance remained significant, *F*(3, 233) = 26.813, *p* < 0.001, as did the post hoc univariate analyses in social support, *F*(1, 479) = 6.625, *p* = 0.01, *η*_p_^2^ = 0.003, less than small effect, attachment insecurity, *F*(1, 258) = 31.549, *p* < 0.001, *η*_p_^2^ = 0.013, small effect, and negative affect, *F*(1, 115) = 71.752, *p* < 0.001, *η*_p_^2^ = 0.030, small effect.

### 3.3 Mediation Effect of Negative Affect Between Interpersonal Problems and NE Symptoms

Effects of the mediation models are shown in Table 2. The total model of social support predicting NE symptoms mediated by negative affect was significant, with *F*(1, 238) = 163.033, *p* < 0.001, *R* ^2^ = 0.07, small effect. After including BMI as a covariate, the mediation remained significant, with *F*(2, 237) = 95.361, *p* < 0.001, *R* ^2^ = 0.09, small effect. Furthermore, the total model of attachment insecurity predicting NE symptoms mediated by negative affect also revealed a significant effect, with *F*(1, 235) = 227.642, *p* < 0.001, *R* ^2^ = 0.10, small effect. Again, after controlling for BMI, the mediation revealed a significant effect, with *F*(2, 234) = 129.88, *p* < 0.001, *R* ^2^ = 0.12, small effect.

**TABLE 2:** Direct and indirect effects of the mediation models of negative affect on interpersonal problems and NE symptoms.

Path	Independent variable	Dependent variable	*t*	Direct effect [95% CI]	Indirect effect [95% CI]
Social support					
a	F‐SozU K‐6	PHQ‐4	−10.649	−0.13 [−0.15, −0.10]	
b	PHQ‐4	NEQ	20.227	0.94 [0.84, 1.03]	
c	F‐SozU K‐6	NEQ	−12.768	−0.27 [−0.31, −0.23]	
c′	F‐SozU K‐6	NEQ	−8.003	−0.15 [−0.19, −0.12]	−0.12 [−0.14, −0.09]
Attachment insecurity					
a	ECR‐RD12	PHQ‐4	16.118	0.77 [0.67, 0.86]	
b	PHQ‐4	NEQ	18.287	0.88 [0.78, 0.97]	
c	ECR‐RD12	NEQ	15.088	1.44 [1.25, 1.63]	
c′	ECR‐RD12	NEQ	8.567	0.77 [0.59, 0.94]	0.67 [0.57, 0.78]

*Note:* All effects are significant at two‐tailed *p* < 0.001.

Abbreviations: ECR‐RD12 = Experiences in Close Relationships Questionnaire, F‐SozU K‐6 = Perceived Social Support Questionnaire, NEQ = Night Eating Questionnaire, PHQ‐4 = Patient Health Questionnaire‐4.

## 4 Discussion

This study uniquely examined the role of interpersonal problems and negative affect in individuals with and without NE symptoms in a large representative German population sample of 2423 individuals. As expected, individuals with NE symptoms reported less social support, more insecure attachment, and greater negative affect than those without NE symptoms. Furthermore, negative affect mediated both the relationships between social support and NE symptoms, and between attachment insecurity and NE symptoms. Effect sizes ranged from less than small to small. All results remained significant after controlling for BMI.

In accordance with the first hypothesis, participants with NE symptoms reported less social support than those without (less than small effect). This finding is consistent with previous research describing lower social support in individuals with symptoms of anorexia nervosa, bulimia nervosa, or BED vs. without in a population sample of 36,309 adults (Kim, Smith, et al. 2023). The onset of NES was linked to stressful life events in a qualitative study of 10 outpatients with NE symptoms (Shillito et al. 2018). Similarly, maladaptive coping mediated the relationship of perceived stress and NE symptoms in 95 college students (Wichianson et al. 2009). These findings suggest that individuals lacking social support, a general resistance resource in stress regulation (Antonovsky et al. 1997), may exhibit NE symptoms as a maladaptive coping strategy in response to stressful life events. Furthermore, the qualitative study by Shillito et al. (2018) highlighted social support as a coping mechanism for NE symptoms. Thus, individuals with greater social support may be better equipped to manage stress, thereby preventing the adoption of maladaptive coping strategies like NE symptoms, or may mitigate the clinical consequences of NES through more effective stress management. While the underlying mechanism remains to be elucidated, this study offers first insight into social support in NE symptoms.

Furthermore, as expected, greater attachment insecurity was found in individuals with NE symptoms vs. without (small effect), aligning with prior research indicating an association between attachment insecurity and NE symptoms in two large population‐based samples of the UK population (Wilkinson et al. 2022) and in a clinical sample of 741 adults (Morse et al. 2006). Since NE symptoms were described as a response to conflictual relationships in a qualitative study of 31 adults with NE symptoms (Cleator et al. 2014), and destructive conflict management was associated with insecure attachment in 405 couples from the population (González‐Ortega et al. 2021), insecure attachment may contribute to the onset of NES. Furthermore, as individuals with insecure attachment anxiety are theorized to exhibit dysfunctional stress responses (Mikulincer and Shaver 2010), the vulnerability to stressful life events may be increased in individuals with insecure attachment, promoting NE symptoms as a maladaptive coping strategy. Moreover, in a qualitative study of 10 adults with NE symptoms, relationship difficulties were proposed as aftereffects of NE symptoms (Shillito et al. 2018), and individuals with NE symptoms vs. without reported more social distress due to nocturnal eating in a population sample of 1514 adults (Fischer et al. 2012). As attachment styles appear to develop during childhood in response to interpersonal conflict (Mikulincer and Shaver 2010), NE symptoms and the resulting interpersonal distress may enhance insecure attachment. In conclusion, this study provides initial evidence of an association between attachment insecurity and NE symptoms, highlighting the need for future research to determine whether attachment insecurity is a risk factor or consequence of NES.

As hypothesized, greater negative affect in adults with NE symptoms vs. without was demonstrated (small effect), consistent with large population‐based studies (Matsui et al. 2021; Kim et al. 2022). Negative affect may influence the development of NES, as in qualitative studies of adults with NE symptoms, individuals with NE symptoms described engaging in NE symptoms in response to negative affect (Cleator et al. 2014), decreasing distress by eating (Shillito et al. 2018), and feeling a temporary release of negative thoughts due to NE symptoms (Latzer et al. 2024). Additionally, 10 outpatients with NE symptoms (Shillito et al. 2018) and 18 treatment‐seeking women with NES (Latzer et al. 2024) reported that NE symptoms in turn led to further distress and negative affect, and Beauchamp et al. (2021) identified mood as a central symptom in NES, indicating that negative affect may also impact the maintenance of NES.

In line with the mediation hypothesis, this study provided first evidence for a cross‐sectional mediational effect of negative affect on the relationship between interpersonal problems and NE symptoms (small effect). Thus, interpersonal problems are associated with NE symptoms particularly in individuals with greater negative affect. A similar cross‐sectional mediation effect has been repeatedly described in other EDs (Ivanova et al. 2015; Karam et al. 2020) and proposed in etiological models (Rieger et al. 2010; Wilfley et al. 2000), which form the theoretical foundation of interpersonal psychotherapy (IPT; Karam et al. 2019). Furthermore, in qualitative studies with adults exhibiting NE symptoms, emotion regulation difficulties were proposed to maintain NE symptoms (Shillito et al. 2018), with dissociation from difficult emotions (Latzer et al. 2024). Given the elevated negative emotions in individuals with vs. without NE symptoms (Matsui et al. 2021; Kim et al. 2022), individuals with NE symptoms may be prone to emotional eating, which is defined as eating in response to negative emotions (Ganley 1989). Empirical evidence further supports this association, as emotional and uncontrolled eating have been associated with NE symptoms in a clinical sample (Morse et al. 2006) and in veterans (Dorflinger et al. 2017) and mediated cross‐sectionally the association between attachment insecurity and NE symptoms in a population‐based study of 486 individuals (Wilkinson et al. 2022). Thus, a deficiency in emotion regulation may favor the utilization of maladaptive coping strategies, such as NE symptoms, in response to negative affect.

Among the strengths of this study is the large sample of the German population, representative regarding age, sex, and education, permitting the generalization of findings to Western industrialized countries. Compared to data of the German Federal Statistical Office from the survey year, this study sample included marginally more female participants (53.5% vs. 51.02%; Federal Statistical Office 2024b) and individuals with a slightly older mean age (50.82 vs. 44.2 years; Federal Statistical Office 2024a). Despite many precautions to avoid sampling biases, this sample was not representative of the German general population in terms of education, with a lower proportion of individuals with ≥ 12 years of school (18.7% vs. 27.9%; Federal Statistical Office 2020) and regarding the prevalence of obesity (9.7% vs. 15.7%; Federal Statistical Office 2024c). The latter is common in population‐based studies with self‐reported BMI, as a trend of underreporting weight and overreporting height compared to measurement was found in a systematic review of 64 studies (Connor Gorber et al. 2007). Further, well‐established instruments for the assessment of NE symptoms, social support, attachment insecurity, and negative affect were employed. As the internal consistency of the NEQ is unsatisfactory and the positive predictive value (PPV) of cutoff ≥ 25 is only 40.7% (Allison, Lundgren, et al. 2008), further investigation into assessment tools for NE symptoms seems to be warranted. Limitations of this study include the small sample size of individuals with NE symptoms decreasing statistical power. Moreover, the response rate of this study (57.9%) may have biased the representativeness of the sample, although it is typical for general population studies (German National Cohort (GNC) Consortium 2014). It should be further taken into consideration that the NEQ (Allison, Lundgren, et al. 2008) includes two mood items, overlapping conceptually with the PHQ‐4 (Löwe et al. 2010). Additionally, mediation analyses via PROCESS macro by Hayes (2018) do not allow conclusions regarding whether negative affect is a partial or full mediator (Meule 2019).

This study uniquely found that individuals exhibiting NE symptoms reported lower social support, greater attachment insecurity, and more negative affect, with negative affect mediating the associations between both social support and attachment insecurity with NES. All results remained significant when using BMI as a covariate, confirming that the described interpersonal problems and mediational effect stem from the NE symptoms and not from higher BMI in individuals with vs. without NE symptoms (see Table 1). Despite less than small and small effect sizes due to limited construct assessment and therefore limited clinical implications, this research provides an essential first step towards understanding potential risk factors for NES by identifying these cross‐sectional relationships between interpersonal problems and NES in a population‐based sample (Jacobi et al. 2004; Kraemer et al. 1997). In this study, the NEQ cutoff ≥ 25 was chosen to determine NE symptoms to preserve statistical power, as the utilization of the NEQ cutoff ≥ 30 reduced the sample size of individuals with NE symptoms to *n* = 8 (see Table S5 in the Supporting Information material). However, future research may adopt the NEQ cutoff ≥ 30 for its greater PPV (Allison, Lundgren, et al. 2008). Considering the cross‐sectional design of this study, further longitudinal research, particularly regarding the mediational effect, is required to clarify the temporal dynamics of these relationships. If confirmed as risk or maintaining factors, and after validation in clinical samples, considering interpersonal factors and negative affect in the treatment of NES may be crucial. Given the efficacy of IPT in treating EDs (Miniati et al. 2018), this therapeutic approach may be explored as a psychological therapy for individuals with a diagnosis of NES to reduce NE symptoms, associated interpersonal problems, and negative affect, and improve distress and quality of life.

## Author Contributions

**Charlotte P. H. Rottschäfer:** conceptualization, methodology, formal analysis, data curation, writing – original draft, writing – review and editing, visualization. **Danielle Schewe:** conceptualization, methodology, formal analysis, writing – review and editing, supervision. **Martina de Zwaan:** funding acquisition, writing – review and editing. **Bernhard Strauss:** funding acquisition, writing – review and editing. **Elmar Brähler:** writing – review and editing, funding acquisition. **Anja Hilbert:** conceptualization, methodology, investigation, resources, writing – review and editing, funding acquisition, supervision, project administration.

## Ethics Statement

The study was approved by the Ethics Committee of the Medical Faculty of the University of Leipzig (number: 050/13‐03.05.2013).

## Conflicts of Interest

Dr. Hilbert reports receiving research grants from the German Federal Ministry of Education and Research, German Research Foundation, Innovation Fund, and Roland Ernst Foundation for Health Care; royalties for books on the treatment of eating disorders and obesity with Hogrefe and Kohlhammer; honoraria for workshops and lectures on eating disorders and obesity and their treatment, including from Lilly and Novo Nordisk; honoraria as editor of the *International Journal of Eating Disorders*; honoraria as a reviewer from Oxford University Press and the German Society for Nutrition; and honoraria as a consultant for Takeda. Dr. de Zwaan reports receiving honoraria from Novo Nordisk, Chiesi, and Novartis and authoring articles and books published by Springer, Routledge, and Elsevier. No other conflicts of interest are declared.

## Supporting information


**Data S1.** Supporting Information.

## Data Availability

The data that support the findings of this study are available on request from senior author A.H. The data are not publicly available due to privacy or ethical restrictions.
