# Supplementary material for: Associations Between Interpersonal Problems, Negative Affect, and Symptoms of Night Eating Syndrome
Source: Int J Eat Disord. 2025 May 19;58(8):1572–81. doi: 10.1002/eat.24463 (PMC12336760; doi:10.1002/eat.24463)
Supplement: Supplementary file 1 — Data S1. Supporting Information. [file EAT-58-1572-s001.docx]

**Supplementary Material for:**

Charlotte P. H. Rottschäfer, Danielle Schewe, Martina de Zwaan, Bernhard Strauss, Elmar Brähler, and Anja Hilbert

Associations Between Interpersonal Problems, Negative Affect, and Symptoms of Night Eating Syndrome

**Supplementary Tables**

**Supplementary Table S1.** List of Questionnaires used in the Original Study

**Supplementary Table S2.** List of Sociodemographic and Anthropometric Variables assessed in the Original Study

**Supplementary Table S3.** Sociodemographic and Descriptive Statistics of Participants aged < 18 years

**Supplementary Table S4.** Spearman Correlation Analyses between NE Symptoms, Interpersonal Problems, Negative Affect, and Sociodemographics of Participants aged < 18 years

**Supplementary Table S5.** Sociodemographic and Descriptive Statistics by NEQ cutoff ≥ 30

**Supplementary Table S6.** Spearman Correlation Analyses between NE Symptoms, Interpersonal Problems, Negative Affect, and Sociodemographics

**Supplementary Text**

**Group Differences in Participants With and Without NE Symptoms by NEQ cutoff ≥ 30**

**Supplementary References**

**Supplementary Methods**

**Supplementary Table S1**

*List of Questionnaires used in the Original Study*

| Instrument | Psychometric Studies |
| --- | --- |
| Night Eating Questionnaire (NEQ) | Allison et al., 2008; Meule et al., 2014 |
| Eating Disorder Examination – Questionnaire 8 (EDE-Q8) | Fairburn & Beglin, 1994; Kliem et al., 2016 |
| Eating Attitudes Test-13 (EAT-13) | Berger et al., 2012; Garner et al., 1982 |
| Perceived Social Support Questionnaire (F-SozU K-6) | Kliem et al., 2015; Sommer & Fydrich, 1991 |
| Experience in Close Relationship Questionnaire Revised Deutsch (ECR-RD12) | Brenk-Franz et al., 2018; Brennan et al., 1998 |
| Patient Health Questionnaire-4 (PHQ-4) | Löwe et al., 2010; Wicke et al., 2022 |
| Giessen Subjective Complaints List (GBB_8) | Brähler et al., 2008; Kliem et al., 2017 |
| Chronic Pain Grade questionnaire (GPG) | Klasen et al., 2004; Von Korff, 1992 |
| Self-Administered Comorbidity Questionnaire German (SCQ-D) | Sangha et al., 2003; Streibelt et al., 2012 |
| The German Health Survey-1997/98 Short Version (BGS98-kurz) | Bellach et al., 1998 |
| Patient-Doctor-Relationship-Questionnaire (PDRQ-9) | Van der Feltz-Cornelis et al., 2004; Zenger et al., 2014 |
| Perceived Autonomy (PA) | Schwarzer, 2008; Warner et al., 2011 |
| Self-Regulation Scale (SRS) | Diehl et al., 2006; Schwarzer et al., 1999 |
| Brief Resilient Coping Scale (BRCS) | Chmitorz et al., 2018; Sinclair & Wallston, 2004 |
| Emotion Regulation Questionnaire (ERQ-4) | Abler & Kessler, 2009; Gross & John, 2003 |
| Erziehungsfragebogen (EFB-8) | Arnold et al., 1993; Naumann et al., 2010 |
| German Fatigue Severity Scale (FSS) | Krupp et al., 1989; Reske et al., 2006 |
| Toronto Alexithymia Scale (TAS-6) | Bach et al., 1996; Bagby et al., 1994 |
| Operationalisierte Psychodynamische Diagnostik – Körperliches Wohlbefinden (OPD-SFK) | Ehrenthal et al., 2012; Ehrenthal et al., 2015 |
| Inventory of Personality Organisation (IPO-16) | Zimmermann et al., 2013 |
| Dysmorphic Concern Questionnaire (DCQ) | Mancuso et al., 2010; Oosthuizen et al., 1998 |
| Child Trauma Questionnaire – short version (CTQ) | Bernstein & Fink, 1998; Grabe et al., 2012 |

**Supplementary Table S2**

*List of Sociodemographic and Anthropometric Variables assessed in the Original Study.*

| Variable | Unit / Response Options |
| --- | --- |
| Age | years |
| Sex | male / female |
| Nationality | German / not German |
| If “not German” | Egyptian / Algerian / Bangladeshi / British / Bulgarian  Algerian / Bangladeshi / British / Bulgarian /  French / Greek / Indian / Iranian / Italian / Kosovar /  Croatian / Latvian / Moroccan / Dutch / Austrian /  Polish / Romanian / Russian / Swiss / Spanish / Thai /  Czech / Turkish / Hungarian / American / Vietamnese |
| Family status | single /  married, living together /  married, not living together /  divorced /  widowed |
| Employment status | employed ≥ 35h/week /  employed 15 – 34h/week /  employed on hourly basis /  military service, civil service, maternity or parental leave /  unemployed, furlough /  retiree /  homemaker /  vocational training /  in school, in university |
| Number of Individuals in the household |  |
| Number of own children |  |
| Number of children in the household |  |
| Own income | < 500 €/month / 500 - < 650€/month /  650 – < 750€/month / 750 - < 900€/month /  900 - < 1000€/month / 1000 - < 1150€/month/  1150 - < 1250€/month / 1250 - < 1500€/month /  1500 – < 2000€/month / 2000 - < 2500€/month /  2500 - < 3500€/month / 3500 - < 5000€/month /  ≥ 5000€/month |
| Household net income | < 500 €/month / 500 - < 650€/month /  650 – < 750€/month / 750 - < 900€/month /  900 - < 1000€/month / 1000 - < 1150€/month/  1150 - < 1250€/month / 1250 - < 1500€/month /  1500 – < 2000€/month / 2000 - < 2500€/month /  2500 - < 3500€/month / 3500 - < 5000€/month /  ≥ 5000€/month |
| Area size (BIK classification) | < 2000 inhabitants /  2000 – < 5000 inhabitants /  5000 - < 20000 inhabitants /  20000 - < 50000 inhabitants /  50000 - < 100000 inhabitants /  100000 – < 500000 inhabitants /  ≥ 500000 inhabitants |
| Area size (political) | < 2000 inhabitants /  2000 – < 5000 inhabitants /  5000 - < 20000 inhabitants /  20000 - < 50000 inhabitants /  50000 - < 100000 inhabitants /  100000 – < 500000 inhabitants /  ≥ 500000 inhabitants |
| Height | meter |
| Weight | kilogram |

**Supplementary Results**

**Supplementary Table S3**

*Sociodemographic and Descriptive Statistics of Participants aged < 18 years*

| Sociodemographics | Missing values | Total sample *N* = 83 | |
| --- | --- | --- | --- |
|  | *n* | *n* | % |
| Sex, female | 0 | 36 | 43.4 |
| Nationality  German  Not German | 0 | 82  1 | 98.8  1.2 |
| Years of school education  ≥ 12 years | 3 | 0 | 0 |
| Employment status  Employed ≥ 35h/week  Employed 15 - 34h/week  Unemployed  Vocational training  School, University | 0 | 0  1  1  7  74 | 0  1.2  1.2  8.4  89.2 |
| Household income, €/month  Low, < 900  Medium, 900 - 1999  High, ≥ 2000 | 5 | 3  10  65 | 3.6  12.0  78.3 |
| Family status  Single  Married/living together  Married/not living together  Divorced  Widowed | 0 | 83  0  0  0  0 | 100  0  0  0  0 |
| Weight status, kg/m^2^  Underweight, < 18,5  Normal weight, 18,5 - 24,9  Overweight, 25 - 29,9  Obesity, ≥ 30 | 1 | 5  69  4  4 | 6.0  83.1  4.8  4.8 |
| NEQ cutoff ≥ 25, 0 - 52 | 0 | 1 | 1.2 |
| NEQ cutoff ≥ 30, 0 - 52 | 0 | 0 | 0 |
|  |  | *M* | *SD* |
| Age, years | 0 | 15.69 | 1.12 |
| BMI, kg/m^2^ | 1 | 22.53 | 3.46 |
| NEQ, 0 - 52 | 0 | 8.84 | 4.59 |
| F-SozU K-6, 1 - 30 | 1 | 26.57 | 2.95 |
| ECR-RD12, 1 - 7 | 8 | 2.92 | 0.86 |
| PHQ-4, 0 - 12 | 1 | 5.18 | 1.50 |

*Note.* Due to missing data, values may not sum up to 100%. The participant scoring NEQ ≥ 25 reported evening hyperphagia, defined by scoring > 1 in item 5 of NEQ, representing > 25% of daily intake after dinner. BMI = body mass index (kg/m^2^), ECR-RD12 = Experiences in Close Relationships Questionnaire, F-SozU K-6 = Perceived Social Support Questionnaire, NEQ = Night Eating Questionnaire, PHQ-4 = Patient Health Questionnaire.

**Supplementary Table S4**

*Spearman Correlation Analyses between NE Symptoms, Interpersonal Problems, Negative Affect, and Sociodemographics of Participants aged < 18 years*

|  | 1 | 2 | 3 | 4 | 5 | 6 | 7 | 8 |
| --- | --- | --- | --- | --- | --- | --- | --- | --- |
| 1 NEQ | 1 |  |  |  |  |  |  |  |
| 2 F-SozU K-6 | -.19 | 1 |  |  |  |  |  |  |
| 3 ECR-RD12 | .24 | -.32* | 1 |  |  |  |  |  |
| 4 PHQ-4 | .35* | -.03 | .11 | 1 |  |  |  |  |
| 5 Age | .24 | -.06 | .13 | .03 | 1 |  |  |  |
| 6 Sex ^a^ | .13 | .12 | -.07 | .12 | .10 | 1 |  |  |
| 7 Nationality ^b^ | .02 | .16 | -.12 | .18 | .14 | -13 | 1 |  |
| 8 BMI | .02 | -.21 | .15 | -.12 | .26 | -.30* | -.08 | 1 |

*Note.* No Spearman correlation analyses were possible with the variable education as all participants reported < 12 years of school education. BMI = body mass index (kg/m^2^), ECR-RD12 = Experiences in Close Relationships Questionnaire (1 - 7), F-SozU K-6 = Perceived Social Support Questionnaire (1 - 30), NEQ = Night Eating Questionnaire (0 - 52), PHQ-4 = Patient Health Questionnaire (0 - 12).

^a^ 0 = male, 1 = female. ^b^ 0 = German, 1 = not German.

* *p* = .0018 using Bonferroni correction.

**Supplementary Table S5**

*Sociodemographic and Descriptive Statistics by NEQ cutoff ≥ 30*

| Sociodemographics | Missing values | NE symptoms *n* = 8 | | No NE symptoms *n* = 2414 | |  | | |
| --- | --- | --- | --- | --- | --- | --- | --- | --- |
|  | *n* | *n* | % | *n* | % | *U / χ²* | *Z / φ* | *p* |
| Sex, female | 0 | 6 | 75.0 | 1129 | 53.4 | 1.49 | .03 | .22 |
| Nationality  German  Not German | 0 | 8  0 | 100  0 | 2320  94 | 96.1  3.9 | 0.32 | -.01 | .57 |
| Years of school education  ≥ 12 years | 7 | 1 | 12.5 | 453 | 18.8 | 0.21 | -.01 | .65 |
| Household income, €/month  Low, < 900  Medium, 900 - 1999  High, ≥ 2000 | 71 | 2  4  2 | 25.0  50.0  25.0 | 180  1051  1112 | 7.5  43.5  46.1 | 6576.00 | -1.63 | .10 |
| Family status  Single  Married/living together  Married/not living together  Divorced  Widowed | 0 | 2  3  0  2  1 | 25.0  37.5  0  25.0  12.5 | 619  1109  63  349  274 | 25.6  45.9  2.6  14.5  11.4 | 0.96 | .02 | .92 |
| Weight status, kg/m^2^  Underweight, < 18,5  Normal weight, 18,5 - 24,9  Overweight, 25 - 29,9  Obesity, ≥ 30 | 14 | 0  1  3  4 | 0  12.5  37.5  50.0 | 19  1161  991  229 | 0.8  48.4  41.3  9.5 | 4432.00 | -2.91 | .004 |
|  |  | *M* | *SD* | *M* | *SD* | *U* | *Z* | *p* |
| Age, years | 0 | 44.63 | 11.21 | 50.84 | 17.51 | 7600.50 | -1.04 | .298 |
| BMI, kg/m^2^ | 14 | 31.53 | 8.29 | 25.29 | 3.76 | 3880.50 | -2.91 | .004 |
| F-SozU K-6, 1 - 30 | 12 | 21.86 | 3.13 | 23.98 | 4.58 | 5467.00 | -1.61 | .11 |
| ECR-RD12, 1 - 7 | 43 | 3.87 | 0.97 | 2.49 | 1.10 | 3160.50 | -3.26 | .001 |
| PHQ-4, 0 - 12 | 29 | 9.00 | 2.88 | 5.74 | 2.29 | 3612.00 | -3.18 | .001 |

*Note.* The NEQ cutoff categorized NE symptoms (≥ 30) and No NE symptoms (< 30). In individuals with NE symptoms, 4 (50.0%) individuals reported evening hyperphagia and 2 (25.0%) individuals described nocturnal ingestion, of which one (12.5%) individual described both. Evening hyperphagia was defined by scoring > 1 in item 5, representing > 25% of daily intake after dinner, and nocturnal ingestion by scoring > 1 in item 12, indicating eating during at least half of the nocturnal awakenings. NEQ = Night Eating Questionnaire (0 - 52). Due to missing data, values may not sum up to 100%. Mann-Whitney U tests indicated differences between individuals with versus without NE symptoms in ordinal and metric variables, whereas chi-squared test investigated group differences in dichotomic and nominal variables. The significance level for these tests was α = .004 using Bonferroni correction. BMI = body mass index (kg/m^2^), ECR-RD12 = Experiences in Close Relationships Questionnaire, F-SozU K-6 = Perceived Social Support Questionnaire, NES = night eating syndrome, PHQ-4 = Patient Health Questionnaire.

**Supplementary Table S6**

*Spearman Correlation Analyses between NE Symptoms, Interpersonal Problems, Negative Affect, and Sociodemographics*

|  | 1 | 2 | 3 | 4 | 5 | 6 | 7 | 8 | 9 |
| --- | --- | --- | --- | --- | --- | --- | --- | --- | --- |
| 1 NEQ | 1 |  |  |  |  |  |  |  |  |
| 2 F-SozU K-6 | -.25* | 1 |  |  |  |  |  |  |  |
| 3 ECR-RD12 | .32* | -.49* | 1 |  |  |  |  |  |  |
| 4 PHQ-4 | .45* | -.25* | .33* | 1 |  |  |  |  |  |
| 5 Age | .02 | -.10* | -.05* | .16* | 1 |  |  |  |  |
| 6 Sex ^a^ | .10* | .03 | .04 | .12* | .01 | 1 |  |  |  |
| 7 Nationality ^b^ | -.02 | -.02 | .02 | .03 | -.09* | -.03 | 1 |  |  |
| 8 Education ^c^ | -.07* | .05* | -.08* | -.06* | -.15* | -.06* | .03 | 1 |  |
| 9 BMI | .07* | .00 | -.04 | .00 | .24* | -.18* | -.06 | -.10* | 1 |

*Note.* BMI = body mass index (kg/m^2^), ECR-RD12 = Experiences in Close Relationships Questionnaire (1 - 7), F-SozU K-6 = Perceived Social Support Questionnaire (1 - 30), NEQ = Night Eating Questionnaire (0 - 52), PHQ-4 = Patient Health Questionnaire (0 - 12).

^a^ 0 = male, 1 = female. ^b^ 0 = German, 1 = not German. ^c^ 0 = < 12 years of school, 1 = ≥ 12 years of school.

* *p* = .0014 using Bonferroni correction.

**Supplementary Text**

**Group Differences in Participants With and Without NE Symptoms by NEQ cutoff ≥ 30**

There was a significant multivariate effect in individuals with versus without NE symptoms on social support, attachment insecurity, and negative affect, *F*(3, 234) = 7.986, *p* < .001. Post-hoc univariate analyses revealed statistically significant differences between participants with and without NE symptoms in attachment insecurity, *F*(1, 234) = 13.479, *p* < .001, η_p_² = .006, less than small effect, and negative affect, *F*(1, 234) = 17.821, *p* < .001, η_p_² = .008, less than small effect, but no significance between individuals with versus without NE symptoms in social support, *F*(1, 234) = 1.608, *p* = .21. Participants with NE symptoms reported lower social support, more insecure attachment and greater negative affect compared to participants without NE symptoms. After adding BMI as covariate, the multivariate effect remained significant, *F*(3, 233) = 7.886, *p* < .001, as did the post-hoc univariates analyses in attachment insecurity, *F*(1, 233) = 14.010, *p* < .001, η_p_² = .006, less than small effect, and negative affect, *F*(1, 233) = 17.085, *p* < .001, η_p_² = .007, less than small effect. Again, no significant effect was displayed between individuals with versus without NE symptoms in social support, F(1, 233) = 1.763, *p* = .18.

**Supplementary References**

Abler, B., & Kessler, H. (2009). Emotion Regulation Questionnaire–eine deutschsprachige Fassung des ERQ von Gross und John. *Diagnostica*, *55*(3), 144-152. https://doi.org/10.1026/0012-1924.55.3.144

Allison, K. C., Lundgren, J. D., O'Reardon, J. P., Martino, N. S., Sarwer, D. B., Wadden, T. A., Crosby, R. D., Engel, S. G., & Stunkard, A. J. (2008). The Night Eating Questionnaire (NEQ): Psychometric properties of a measure of severity of the night eating syndrome. *Eating Behaviors*, *9*(1), 62-72. <https://doi.org/10.1016/j.eatbeh.2007.03.007>

Arnold, D. S., O'leary, S. G., Wolff, L. S., & Acker, M. M. (1993). The Parenting Scale: A measure of dysfunctional parenting in discipline situations. *Psychological Assessment*, *5*(2), 137-144. <https://psycnet.apa.org/doi/10.1037/1040-3590.5.2.137>

Bach, M., Bach, D., de Zwaan, M., Serim, M., & Böhmer, F. (1996). Validierung der deutschen Version der 20-Item Toronto-Alexithymie-Skala bei Normalpersonen und psychiatrischen Patienten [Validation of the German version of the 20-item Toronto Alexithymia Scale in normal persons and psychiatric patients]. *Psychotherapie, Psychosomatik, Medizinische Psychologie*, *46*(1), 23-28.

Bagby, R. M., Parker, J. D., & Taylor, G. J. (1994). The twenty-item Toronto Alexithymia Scale-I. Item selection and cross-validation of the factor structure. *Journal of Psychosomatic Research*, *38*(1), 23-32. https://doi.org/10.1016/0022-3999(94)90005-1

Bellach, B. M., Knopf, H., & Thefeld, W. (1998). Der Bundes-Gesundheitssurvey. 1997/98 [The German Health Survey. 1997/98]. *Gesundheitswesen*, *60*(Suppl. 2), 59-68.

Berger, U., Hentrich, I., Wick, K., Bormann, B., Brix, C., Sowa, M., Schwartze, D., & Strauß, B. (2012). Eignung des "Eating Attitudes Test" EAT-26D zur Erfassung riskanten Essverhaltens bei 11- bis 13-Jährigen und Vorschlag für eine Kurzversion mit 13 Items [Psychometric quality of the "Eating Attitudes Test" (German version EAT-26D) for measuring disordered eating in pre-adolescents and proposal for a 13-item short version]. *Psychotherapie, Psychosomatik, Medizinische Psychologie*, *62*(6), 223-226. <https://doi.org/10.1055/s-0032-1308994>

Bernstein, D. P., Fink, L. (1998). *Childhood Trauma Questionnaire Manual.* The Psychological Corporation.

Brähler, E., Hinz, A., Scheer, J. (2008): *Gießener Beschwerdebogen GBB-24 Handbuch* (3. er-weiterte Auflage). Huber.

Brenk-Franz, K., Ehrenthal, J., Freund, T., Schneider, N., Strauß, B., Tiesler, F., Schauenburg, H., & Gensichen, J. (2018). Evaluation of the short form of "Experience in Close Relationships" (Revised, German Version "ECR-RD12") – A tool to measure adult attachment in primary care. *PloS ONE, 13*, e0191254. <https://doi.org/10.1371/journal.pone.0191254>

Brennan, K. A., Clark, C., & Shaver, P. (1998). *Self-report measurement of adult attachment: An integrative overview.* Guilford.

Chmitorz, A., Wenzel, M., Stieglitz, R. D., Kunzler, A., Bagusat, C., Helmreich, I., Gerlicher, A., Kampa, M., Kubiak, T., Kalisch, R., Lieb, K., & Tüscher, O. (2018). Population-based validation of a German version of the Brief Resilience Scale. *PloS one*, *13*(2), e0192761. <https://doi.org/10.1371/journal.pone.0192761>

Diehl, M., Semegon, A. B., & Schwarzer, R. (2006). Assessing attention control in goal pursuit: A component of dispositional self-regulation. *Journal of Personality Assessment*, *86*(3), 306-317. <https://doi.org/10.1207/s15327752jpa8603_06>

Ehrenthal, J. C., Dinger, U., Horsch, L., Komo-Lang, M., Klinkerfuss, M., Grande, T., & Schauenburg, H. (2012). Der OPD-Strukturfragebogen (OPD-SF): Erste Ergebnisse zu Reliabilität und Validität [The OPD Structure Questionnaire (OPD-SQ): First results on reliability and validity]. *Psychotherapie, Psychosomatik, Medizinische Psychologie*, *62*(1), 25-32. <https://doi.org/10.1055/s-0031-1295481>

Ehrenthal, J. C., Dinger, U., Schauenburg, H., Horsch, L., Dahlbender, R. W., & Gierk, B. (2015). Entwicklung einer Zwölf-Item-Version des OPD-Strukturfragebogens (OPD-SFK) [Development of a 12-item version of the OPD-structure questionnaire (OPD-SQS)]. *Psychosomatische Medizin und Psychotherapie*, *61*(3), 262-274. <https://doi.org/10.13109/zptm.2015.61.3.262>

Fairburn, C. G., & Beglin, S. J. (1994). Assessment of eating disorders: Interview or self‐report questionnaire?. *International Journal of Eating Disorders*, *16*(4), 363-370.

Garner, D. M., Olmsted, M. P., Bohr, Y., & Garfinkel, P. E. (1982). The Eating Attitudes Test: Psychometric features and clinical correlates. *Psychological Medicine*, *12*(4), 871-878. <https://doi.org/10.1017/s0033291700049163>

Grabe, H., Schulz, A., Schmidt, C. O., Appel, K., Driessen, M., Wingenfeld, K., Barnow, S., Spitzer, C., John, U., Berger, K., Wersching, H., & Freyberg, H. J. (2012). Ein Screeninginstrument für Missbrauch und Vernachlässigung in der Kindheit: Der Childhood Trauma Screener (CTS). *Psychiatrische Praxis, 39*(3), 109-115. <https://doi.org/10.1055/s-0031-1298984>

Gross, J. J., & John, O. P. (2003). Individual differences in two emotion regulation processes: Implications for affect, relationships, and well-being. *Journal of Personality and Social Psychology*, *85*(2), 348-362. <https://doi.org/10.1037/0022-3514.85.2.348>

Klasen, B. W., Hallner, D., Schaub, C., Willburger, R., & Hasenbring, M. (2004). Validation and reliability of the German version of the Chronic Pain Grade Questionnaire in primary care back pain patients. *Psycho-Social Medicine*, *1*, Doc07.

Kliem, S., Lohmann, A., Klatt, T., Mößle, T., Rehbein, F., Hinz, A., Beutel, M., & Brähler, E. (2017). Brief assessment of subjective health complaints: Development, validation and population norms of a brief form of the Giessen Subjective Complaints List (GBB-8). *Journal of Psychosomatic Research*, *95*, 33-43. <https://doi.org/10.1016/j.jpsychores.2017.02.003>

Kliem, S., Mößle, T., Rehbein, F., Hellmann, D. F., Zenger, M., & Brähler, E. (2015). A brief form of the Perceived Social Support Questionnaire (F-SozU) was developed, validated, and standardized. *Journal of Clinical Epidemiology, 68*(5), 551-562. <https://doi.org/10.1016/j.jclinepi.2014.11.003>

Kliem, S., Mößle, T., Zenger, M., Strauß, B., Brähler, E., & Hilbert, A. (2016). The Eating Disorder Examination-Questionnaire 8: A brief measure of eating disorder psychopathology (EDE-Q8). *International Journal of Eating Disorders*, *49*(6), 613-616. <https://doi.org/10.1002/eat.22487>

Krupp, L. B., LaRocca, N. G., Muir-Nash, J., & Steinberg, A. D. (1989). The Fatigue Severity Scale. Application to patients with multiple sclerosis and systemic lupus erythematosus. *Archives of Neurology*, *46*(10), 1121-1123. <https://doi.org/10.1001/archneur.1989.00520460115022>

Löwe, B., Wahl, I., Rose, M., Spitzer, C., Glaesmer, H., Wingenfeld, K., Schneider, A., Brähler, E. (2010). A 4-item measure of depression and anxiety: Validation and standardization of the Patient Health Questionnaire-4 (PHQ-4) in the general population. *Journal of Affective Disorders*, *122*(1-2), 86-95. https://doi.org/10.1016/j.jad.2009.06.019

Mancuso, S. G., Knoesen, N. P., & Castle, D. J. (2010). The Dysmorphic Concern Questionnaire: A screening measure for body dysmorphic disorder. A*ustralian and New Zealand Journal of Psychiatry*, *44*(6), 535-542. <https://doi.org/10.3109/00048671003596055>

Meule, A., Allison, K. C., & Platte, P. (2014). A German version of the Night Eating Questionnaire (NEQ): Psychometric properties and correlates in a student sample. *Eating Behaviors*, *15*(4), 523-527. <https://doi.org/10.1016/j.eatbeh.2014.07.002>

Naumann, S., Bertram, H., Kuschel, A., Heinrichs, N., Hahlweg, K., & Döpfner, M. (2010). Der Erziehungsfragebogen (EFB). *Diagnostica, 56*(3). https://doi.org/10.1026/0012-1924/a000018

Oosthuizen, P., Lambert, T., & Castle, D. J. (1998). Dysmorphic concern: Prevalence and associations with clinical variables. *Australian and New Zealand Journal of Psychiatry*, *32*(1), 129-132. <https://doi.org/10.3109/00048679809062719>

Reske, D., Pukrop, R., Scheinig, K., Haupt, W. F., & Petereit, H. F. (2006). Messbarkeit von Fatigue bei multipler Sklerose mithilfe standardisierter Methoden im deutschsprachigen Raum [Measuring fatigue in patients with multiple sclerosis with standardized methods in German speaking areas]. *Fortschritte der Neurologie-Psychiatrie*, *74*(9), 497-502. <https://doi.org/10.1055/s-2006-932189>

Sangha, O., Stucki, G., Liang, M. H., Fossel, A. H., & Katz, J. N. (2003). The Self-Administered Comorbidity Questionnaire: A new method to assess comorbidity for clinical and health services research. *Arthritis and Rheumatism, 49*(2), 156-163. <https://doi.org/10.1002/art.10993>

Schwarzer, R. (2008). *Perceived autonomy in old age*. <http://userpage.fu-berlin.de/~health/autonomy.htm>.

Schwarzer, R., Diehl, M., Schmitz, G. S. (1999). *Self-Regulation Scale*. http://www.fu-berlin.de/gesund/skalen

Sinclair, V. G., & Wallston, K. A. (2004). The development and psychometric evaluation of the Brief Resilient Coping Scale. *Assessment*, *11*(1), 94-101. <https://doi.org/10.1177/1073191103258144>

Streibelt, M., Schmidt, C., Brünger, M., & Spyra, K. (2012). Komorbidität im Patientenurteil - geht das? Validität eines Instruments zur Selbsteinschätzung der Komorbidität (SCQ-D) [Comorbidity from the patient perspective - Does it work? Validity of a questionnaire on self-estimation of comorbidity (SCQ-D)]. *Der Orthopäde, 41*(4), 303-310. <https://doi.org/10.1007/s00132-012-1901-3>

Sommer, G., & Fydrich, T. (1991). Entwicklung und Überprüfung eines Fragebogens zur sozialen Unterstützung (F-SozU). *Diagnostica, 37*(2), 160-178.

Van der Feltz-Cornelis, C. M., Van Oppen, P., Van Marwijk, H. W., De Beurs, E., & Van Dyck, R. (2004). A Patient-Doctor Relationship Questionnaire (PDRQ-9) in primary care: Development and psychometric evaluation. *General Hospital Psychiatry*, *26*(2), 115-120. <https://doi.org/10.1016/j.genhosppsych.2003.08.010>

Von Korff, M., Ormel, J., Keefe, F. & Dworkin, S. F. (1992). Grading the severity of chronic pain. *Pain, 50*, 133-49. <https://doi.org/10.1016/0304-3959(92)90154-4>

Warner, L. M., Ziegelmann, J., Schuz, B., Wurm, S., Tesch-Römer, C., & Schwarzer, R. (2011). Maintaining autonomy despite multimorbidity: Self-efficacy and the two faces of social support. *European Journal of Ageing, 8*(1), 3-12. <https://doi.org/10.1007/s10433-011-0176-6>

Wicke, F. S., Krakau, L., Löwe, B., Beutel, M. E., & Brähler, E. (2022). Update of the standardization of the Patient Health Questionnaire-4 (PHQ-4) in the general population. *Journal of Affective Disorders, 312*, 310-314. <https://doi.org/10.1016/j.jad.2022.06.054>

Zenger, M., Schaefert, R., van der Feltz-Cornelis, C., Brähler, E., & Häuser, W. (2014). Validation of the Patient-Doctor-Relationship Questionnaire (PDRQ-9) in a representative cross-sectional German population survey. *PloS one*, *9*(3), e91964. <https://doi.org/10.1371/journal.pone.0091964>

Zimmermann, J., Benecke, C., Hörz, S., Rentrop, M., Peham, D., Bock, A., Wallner, T., Schauenburg, H., Frommer, J., Huber, D., Clarkin, J. F., & Dammann, G. (2013). Validierung einer Deutschsprachigen 16-Item-Version des Inventars der Persönlichkeitsorganisation (IPO-16). *Diagnostica*, *59*(1), 3-16. <https://doi.org/10.1026/0012-1924/a000076>
